# Bagging Nearest-Neighbor Prediction independence Test: an efficient method for nonlinear dependence of two continuous variables

**DOI:** 10.1038/s41598-017-12783-9

**Published:** 2017-10-06

**Authors:** Yi Wang, Yi Li, Xiaoyu Liu, Weilin Pu, Xiaofeng Wang, Jiucun Wang, Momiao Xiong, Yin Yao Shugart, Li Jin

**Affiliations:** 1 Ministry of Education Key Laboratory of Contemporary Anthropology, Collaborative Innovation Center for Genetics and Development, School of Life Sciences, Fudan University, Shanghai, China; 2 State Key Laboratory of Genetic Engineering, Collaborative Innovation Center for Genetics and Development, School of Life Sciences, Fudan University, Shanghai, China; 3 Human Genetics Center, School of Public Health, University of Texas Houston Health Sciences Center, Houston, Texas USA; 4 Unit on Statistical Genomics, Division of Intramural Division Programs, National Institute of Mental Health, National Institutes of Health, Bethesda, MD USA

## Abstract

Testing dependence/correlation of two variables is one of the fundamental tasks in statistics. In this work, we proposed an efficient method for nonlinear dependence of two continuous variables (X and Y). We addressed this research question by using BNNPT (Bagging Nearest-Neighbor Prediction independence Test, software available at https://sourceforge.net/projects/bnnpt/). In the BNNPT framework, we first used the value of X to construct a bagging neighborhood structure. We then obtained the out of bag estimator of Y based on the bagging neighborhood structure. The square error was calculated to measure how well Y is predicted by X. Finally, a permutation test was applied to determine the significance of the observed square error. To evaluate the strength of BNNPT compared to seven other methods, we performed extensive simulations to explore the relationship between various methods and compared the false positive rates and statistical power using both simulated and real datasets (Rugao longevity cohort mitochondrial DNA haplogroups and kidney cancer RNA-seq datasets). We concluded that BNNPT is an efficient computational approach to test nonlinear correlation in real world applications.

## Introduction

Dependence is any statistical relationships between two random variables and correlation describes any kind of the statistical relationships including dependence. In practice, correlations can be used to predict any potential relationships of interest. The Pearson correlation coefficient appears to be the most commonly used method for assessing correlation. However, the Pearson correlation is sensitive to linear correlations, while several other methods are more robust to detect the non-linear correlations^1–3^. Testing linear/nonlinear dependence of two variables is one of the fundamental tasks in statistics.

The Pearson correlation (or Pearson’s r), first proposed by Karl Pearson and Francis Galton^4–8^, is a measure of the correlation between two random variables (X and Y). It assigns a value that varies from -1 to 1. The correlation between the two variables is defined as the product of their covariance divided by their standard deviation. Although the Pearson correlation coefficient is often used, the Pearson’s r of sample statistic is not distributionally robust (non-normal distribution)^9^ and its values may be misleading when there are outliers^10,11^.

The Spearman correlation coefficient (or Spearman’s rho) is a nonparametric statistical method of measuring the statistical association between two variables. It evaluates the process during which two variables can be described by monotonic functions. The Spearman correlation coefficient is defined as the Pearson correlation coefficient of the rank variable^12^. The Kendall rank correlation coefficient (or Kendall’s tau coefficient), proposed by Maurice Kendall in 1938, is another nonparametric statistical method to test the correlations of two variables^13^. And there is no assumption made about the distribution of X and Y or (X, Y).

Other commonly used statistical methods to evaluate the correlations of two random variables include distance correlation, Hoeffding’s independence test, maximal information coefficient (MIC), Hilbert-Schmidt Independence Criterion (HSIC) and so on. The distance correlation is a statistical method of measuring statistical dependence of two random variables. The distance correlation coefficient is zero if and only if the two random variables are statistically independent. The distance correlation coefficient was proposed by Gabor J Szekely (2005), which solves the deficiency of Pearson correlation coefficient (Pearson’s r can be zero for dependent variables). When the Pearson correlation coefficient is 0, it indicates linearly irrelevant but does not imply independence, whereas the distance correlation is 0 if and only if the random variables are statistically independent^14,15^. Hoeffding’s independence test, named after Wassily Hoeffding, is a measure of group deviation. Hoeffding derived an unbiased estimate of H, which can be used to test the independence of the two variables. This test can only be applied to continuously distributed dataset. A sample-based version of this measure was discussed under the null distribution^16^. MIC is an established method to measure the linear or non-linear correlation between two random variables. MIC belongs to the nonparametric statistical method based on the maximal information theory^17^. MIC uses binning to apply mutual information to continuous random variables and MIC is an approach for selecting the number of bins and finding a maximum over possible grids. HSIC (Gretton et al. year) was an independence criterion based on the eigen-spectrum of covariance operators in reproducing kernel Hilbert spaces (RKHSs), consisting of an empirical estimate of the Hilbert-Schmidt Independence Criterion^18^. HHG (proposed by Heller et al.) is a powerful test that is applicable to all dimensions, consistent against all alternatives and is easy to implement^19^.

We had previously developed a new algorithm called continuous variance analysis (CANOVA)^20^, the idea came from the analysis of variance (ANOVA) of continuous response with a categorical factor^21^. In the CANOVA framework, we first define a neighborhood of each data point according to its X value, and then calculate the variance of the Y value within the neighborhood, and finally use a permutation test to assess the significance of the observed “within neighborhood variance”^20^. CANOVA is an efficient method in case of non-linear correlation, especially when the function is highly oscillating.

In the current study, we proposed a new nonlinear dependence measure method: Bagging Nearest-Neighbor Prediction independence Test (BNNPT). BNNPT is based on a permutation test of the square error (SE) of bagging nearest neighbor estimator. In pattern recognition, the k-Nearest Neighbors algorithm (or simply k-NN) is a nonparametric approach for classification and regression^22^. The optimal choice of k depends on the distribution of the data. Typically, large k values may reduce the effect of noise on the classification^23^, but the boundaries between the classes are less distinct. The special case where the class is predicted to be the class of the closest training sample (k = 1) is called the nearest neighbor algorithm. Bagging, also known as “bootstrap aggregation”, is an algorithm for machine learning aggregators that aims to improve the stability and accuracy of the machine learning algorithms. Furthermore, it reduces the variance, decreasing the possibility of over-fitting. Bagging is a special case of the model averaging method. On the other hand, it can slightly reduce the performance of stabilization methods, such as K-nearest neighbors^24^.

In the BNNPT framework, we first used the value of X to construct a bagging neighborhood structure. And then, we got the out of bag estimator of Y based on the bagging neighborhood structure. The square error (SE) was calculated to measure how good Y is predicted by X. Finally, a permutation test was applied to detect the significance of the observed square error. We compared the false positive ratio^25^ and statistical power^26^ of BNNPT with seven other common correlation coefficient algorithms in simulation study. Furthermore, we compared their performance in a real Rugao longevity cohort (mitochondrial DNA haplogroups)^27^ and a kidney cancer RNA-seq (transcriptome sequencing) data set^28,29^.

## Methods

### Summary

The main framework of BNNPT is based on a permutation test^30^ of the square error of a bagging nearest neighbor estimator. For two vectors X and Y of length N, we first construct a bagging neighborhood structure based on X only. The neighborhood structure is an index matrix of N rows and K (number of bags) columns. The element X_Neighborhood(i,j)_ is defined as the *jth* bag’s nearest neighbor of X_*i*_, Neighborhood(i,j) ≠ i. The element of Neighborhood(i,j) is sampled as follow: we draw a bag of mtry values from X, and choose the one X_nearest_ that is closest to X_i_, then Neighborhood(i,j) = nearest. When neighborhood structure is available, we were able to construct a bagging nearest neighbor estimator of each Y_i_:

$${\rm{Hi}}\,=\,{\rm{sum}}({{\rm{Y}}}_{{\rm{Neighborhood}}(i,j)},\,j)/{\rm{bags}}$$

The square error SE = ||H-Y||_2_ indicates how well Y is predicted by X. To assess the statistical significance level, a permutation test is conducted using SE as the test statistics. We randomly shuffle Y many times and count the probability that SE_random_ <  = SE, which is reported as the p-value.

Denote ${{SE}}_{{BNNPT}}(X,Y,b,m)$ as the squared root of the residual of bagging nearest neighbor estimator using X as a predictor and Y as response and *b*(*number of bagging*), *m*(*mtry*) as parameters of BNNPT. Our null hypothesis is the following:

$$S{E}_{BNNPT}(X,Y,b,m)=S{E}_{BNNPT}(X,Y^{\prime} ,b,m),Y^{\prime}  \sim Random\,shuffle(Y)$$

### Pseudocode for BNNPT

Input: two data vector X and Y, both are of length N.

Parameter: bags, mtry (default = sqrt(N)), permutations.

**Algorithm:**
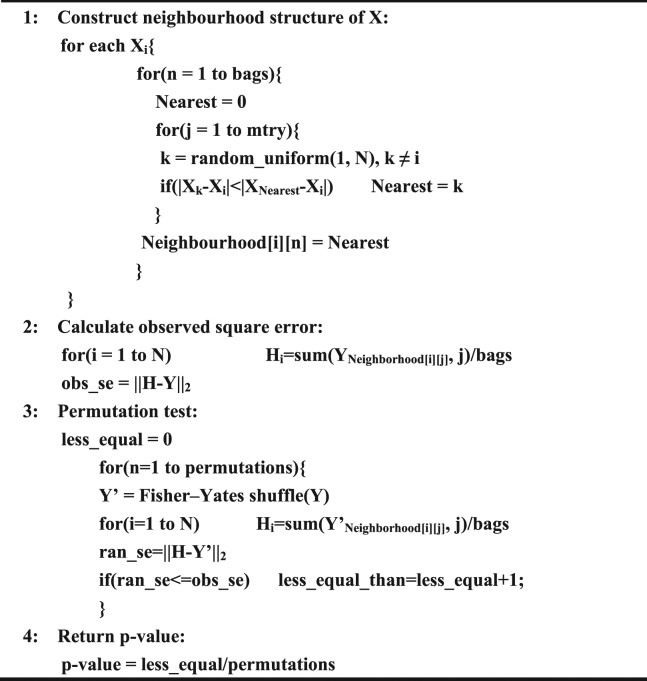
.

### Simulation study

Nine simple functions (including constant function, linear function, quadratic function, sine function and cosine function) were simulated. Additionally, we added Gaussian noise (mean = 0, Gaussian variance = 1) to Y in these nine simple functions, as shown in Table 1. In the simulation data, we set different Gaussian noise levels (mean = 0, Gaussian variance = 1/9, 1/4, 4 and 9) and reported the power across noise levels (shown in Supplemental Materials 1). We selected seven algorithms as the benchmarks: Pearson correlation coefficient, Spearman rank correlation coefficient, Kendall rank correlation coefficient, Distance correlation, Hoeffding’s independence test, MIC and CANOVA. One thousand sets of simulations were carried out to calculate the false positives rate and the statistical power. Two different sample sizes were selected (N = 50 and 760), x as the independent variable which was uniformly distributed in (−1, 1) and y as the dependent variable (shown in Supplemental Materials 1). Notably, MIC has a bias/variance parameter (the ‘alpha’ parameter in the minerva implementation): the maximal allowed resolution of any grid^17^. Reshef et al. also reported that different parameter setting (α = 0.55, c = 5) is faster than the default setting and does not significantly affect performance^31^. For simplicity, the default parameters of the MIC (α = 0.6, c = 15) was used in this work.

**Table 1:** Simulation power in nine sample functions.

N = 50, X ~U(−1,1)	BNNPT	Pearson	Spearman	Kendall	Hoeffding	Distance	CANOVA	MIC
y = 0 + N(0,1)	0.050	0.058	0.053	0.055	0.068	0.059	0.053	0.046
y = x + N(0,1)	0.839	**0.958**	0.951	0.950	0.940	0.946	0.544	0.593
y = $0.5({{\rm{x}}+1)}^{2}$ + N(0,1)	0.861	**0.961**	0.949	0.946	0.935	0.946	0.580	0.608
y = sin($\pi$ x) + N(0,1)	0.957	0.937	0.912	0.904	**0.963**	0.962	0.742	0.805
y = sin(3 $\pi$ x) + N(0,1)	**0.795**	0.180	0.182	0.190	0.201	0.174	0.694	0.423
y = cos($\pi$ x) + N(0,1)	**0.947**	0.066	0.079	0.073	0.690	0.653	0.726	0.649
y = cos(2 $\pi$ x) + N(0,1)	**0.888**	0.060	0.065	0.066	0.151	0.109	0.720	0.570
y = cos(3 $\pi$ x) + N(0,1)	**0.707**	0.064	0.072	0.070	0.109	0.093	0.688	0.394

The bold means the first place result of all methods compared.

### Applications on Rugao longevity cohort dataset

We compared the BNNPT algorithm with the other seven algorithms using a real Rugao longevity cohort for mitochondrial DNA haplogroups, which included 1852 samples (463 exceptional longevity samples, 926 elder sampled, 463 middle-aged samples) and 28 major mitochondrial haplogroups^27^. The samples with missing values were omitted (remained 1835 samples).

The level of correlations between genotype data X (28 mitochondrial haplogroups data) and phenotype data Y (ages) were tested. For simplicity, the other algorithms were applied the default parameters (especially for MIC, α = 0.6, c = 15). The p value results and comparisons are shown in Table 2. The significance level was preset to be 0.05.

**Table 2:** The p-value comparison of benchmarked methods in Rugao longevity cohort data.

mtDNA haplogroup	BNNPT	Pearson	Spearman	Kendall	Hoeffding*	Distance	Canova
D	0.998	0.423	0.567	0.567	1.000	0.421	0.541
D4	0.655	0.175	0.358	0.357	1.000	0.162	0.486
D4a	0.519	0.809	0.888	0.888	1.000	0.951	0.485
D4b	0.568	0.647	0.784	0.784	1.000	0.786	0.482
D4b2	0.981	0.376	0.449	0.449	1.000	0.419	0.508
D4b2b	0.799	0.580	0.548	0.548	1.000	0.728	0.426
D5	0.188	0.568	0.694	0.694	1.000	0.782	0.502
M12	0.907	0.739	0.605	0.605	1.000	0.888	0.527
G	0.303	0.933	0.723	0.723	1.000	0.943	0.507
G2	0.149	0.161	0.232	0.232	1.000	0.261	0.529
M7	0.957	0.961	0.994	0.994	1.000	0.947	0.500
M7b	0.619	0.705	0.992	0.992	1.000	0.806	0.512
M8	0.963	0.863	0.851	0.851	1.000	0.368	0.528
M8a	0.447	0.397	0.365	0.365	1.000	0.146	0.455
C	0.246	0.513	0.583	0.583	1.000	0.713	0.501
M9	0.541	**0.030**	0.054	0.054	1.000	**0.016**	0.433
M10	0.347	0.793	0.963	0.963	1.000	0.866	0.503
N9	0.313	**0.024**	0.060	0.060	1.000	**0.037**	0.435
N9a	0.352	0.084	0.193	0.193	1.000	0.130	0.471
A	**0.029**	0.371	0.530	0.530	1.000	0.532	0.484
F	0.224	0.113	0.065	0.065	1.000	0.170	0.434
F1	0.442	0.239	0.127	0.127	1.000	0.280	0.466
B	0.180	0.388	0.368	0.368	1.000	0.451	0.544
B5	0.656	0.201	0.524	0.524	1.000	0.188	0.501
B5a	0.321	0.189	0.653	0.653	1.000	0.177	0.547
B5b	0.709	0.654	0.740	0.740	1.000	0.479	0.508
B4a	**0.012**	0.097	0.086	0.086	1.000	0.109	0.499
B4b	0.746	0.540	0.833	0.833	1.000	0.544	0.391

The significant (significance level = 0.05) pvalues of methods were marked in bold.

^*^The genotype data X (28 mitochondrial haplogroups data) were drawn from a discontinuous distribution, Hoeffding’s independence test may have a defect for discontinuous distributions.

### Applications on kidney cancer dataset

We also compared the BNNPT algorithm with the other seven algorithms using a real RNA-seq dataset for kidney cancer, which included 604 samples (532 cancer samples, 72 normal samples) and 20531 genes^28,29^.

The level of correlations between genotype data X (20,531 gene expression data) and phenotype data Y (whether kidney cancer or not) were evaluated. The computing time of each algorithm was also compared. The significance level is preset to be 2.435e-06 (Bonferroni correction). For simplicity, the other algorithms were applied the default parameters (especially for MIC, α = 0.6, c = 15). The results and comparisons are shown in Table 3.

**Table 3:** Comparison of computing time and detected significant genes numbers of all methods in kidney cancer dataset (the significance level α = 2.435e-06).

Kidney cancer dataset	BNNPT	Pearson	Spearman	Kendall	Hoeffding	Distance	CANOVA	MIC
The number of unique genes (reported in pubmed)	**387 (15)**	15 (1)	41 (1)	0 (0)	0 (0)	120 (1)	8 (1)	3 (0)
Significant number	10617	8239	**11629**	11569	4953	10946	5901	8081
Computing time (seconds)	80*	**0.0023**	0.0025	0.0082	1.8	~5000	20	0.027

^*^In order to compare the computing time, the number of permutations of BNNPT is set to 10,000,000 times. If the number of permutations of BNNPT is set to 100,000 times, it only needs 1 seconds. The bold means the first place results of all methods compared. The Computing time was recorded between 1 gene and 604 samples.

## Results

### Results from simulation study

It can be seen that when the constant function (y = 0) was used, we compared the false positive rate of the different methods at the significance level of 0.05 in Table 1. Pearson correlation coefficient, Spearman’s rank correlation coefficient, Kendall’s rank correlation coefficient, Distance correlation coefficient, CANOVA, MIC and BNNPT, all showed a false positive rate around 0.05. It does mean that the Type I error rate was adequately controlled. However, the false positive rate of the Hoeffding’s independence test was slightly higher than 0.05. Therefore, it is crucial to note that under settings similar to the simulation study, Hoeffding’s method led to more false positives than the other methods.

For the comparison of the statistical power of other non-constant functions in the simulation data, we observed the following in Table 1: (1) In case of linear correlations, the Pearson correlation coefficient is the most powerful method, BNNPT is less powerful than Pearson correlation coefficient, but does not fail (power > 0.5); (2) In the case of non-linear correlation, BNNPT appeared to be most powerful, when the function is highly oscillatory/nonlinear, its power is higher than other methods. (3) BNNPT is more powerful than the MIC algorithm in all cases.

By comparing the non-constant correlations shown in Supplemental Materials 1: We concluded that: (1) When the Gaussian noise level is low (Gaussian variance = 1/9, 1/4), most of the methods have a high power, especially in simple linear relationships. But BNNPT has a higher power in most non-constant functions, especially in non-linear functions. (2) When the Gaussian noise level is high (e.g. Gaussian variance = 4, 9), most methods had much lower power while BNNPT achieved better power than other methods in complex sine/cosine functions. (3) When the sample size is larger (N = 760), BNNPT still achieved better power than other methods in complex sine/cosine functions. However, Pearson’s correlation coefficient is more powerful in the simple linear functions. Therefore, when the relationship between the two random variables is linear, we recommend the use of the Pearson correlation coefficient to obtain higher statistical power. When the relationship is nonlinear or complicated, BNNPT is a good choice to explore the correlation structure of the data.

### Results from the Rugao longevity cohort dataset

The p-value comparison for the Rugao longevity cohort^27^ is shown in Table 2. It indicated that BNNPT detected two mtDNA haplogroups, haplogroup A and haplogroup B4a (P value < 0.05). Pearson correlation coefficient detected two mtDNA haplogroups: haplogroup M9 and haplogroup N9 (Pvalue < 0.05). Distance also detected two mtDNA haplogroups, the same two as Pearson. All BNNPT and CANOVA results were realized in the C +  + ^32^ environment and the other six benchmarks were calculated using the R packages ‘energy’^33^, ‘Hmisc’^34^ and ‘minerva’^35^. All BNNPT results were calculated in parallel (fully using all 8 CPU cores) on a desktop PC, equipped with an AMD FX-8320 CPU and 32 GB memory. In addition, all of the R code was computed in parallel through an R package named ‘snow’^36^.

Literature review for validation of each haplogroup was then performed in the pubmed database. In one Japanese population, the mitochondrial haplogroups A confers a significant risk for coronary atherosclerosis which is a kind of age-related disease^37^. B4a was reported that has negatively correlated with ages in Rugao population^27^. Haplogroup M9 and haplogroup N9 were reported to be related to longevity^27,38^.

### Results from the kidney cancer study

The comparison and computing time for kidney cancer dataset^28,29^ is shown in Table 3. In order to compare the computing time, the number of permutations of BNNPT is set to be 10,000,000 times (Table 3). In Supplemental Materials 2, we provided genes that were only detected by the BNNPT method (that was not detected by other methods). For comparison, we also listed genes that can only be detected by other methods in Supplemental Materials 3. All BNNPT and CANOVA analyses were conducted in the C +  + ^32^ environment and the other six benchmarks were calculated using the R packages^33–36^.

We observed that the Spearman correlation coefficient can detect the most number of significant genes (11629 genes, α = 2.435e-06, in Table 3) in real kidney cancer RNA-seq data. The BNNPT method detects slightly less (10617 genes) than Spearman’s correlation coefficient. To explore the biological relevance of the detected genes and to compare the features of each method, we use the “uniquely significant genes” detected from each method as the target gene set, and then performed a literature review for validation of each gene in the pubmed database.

**Figure 1:**
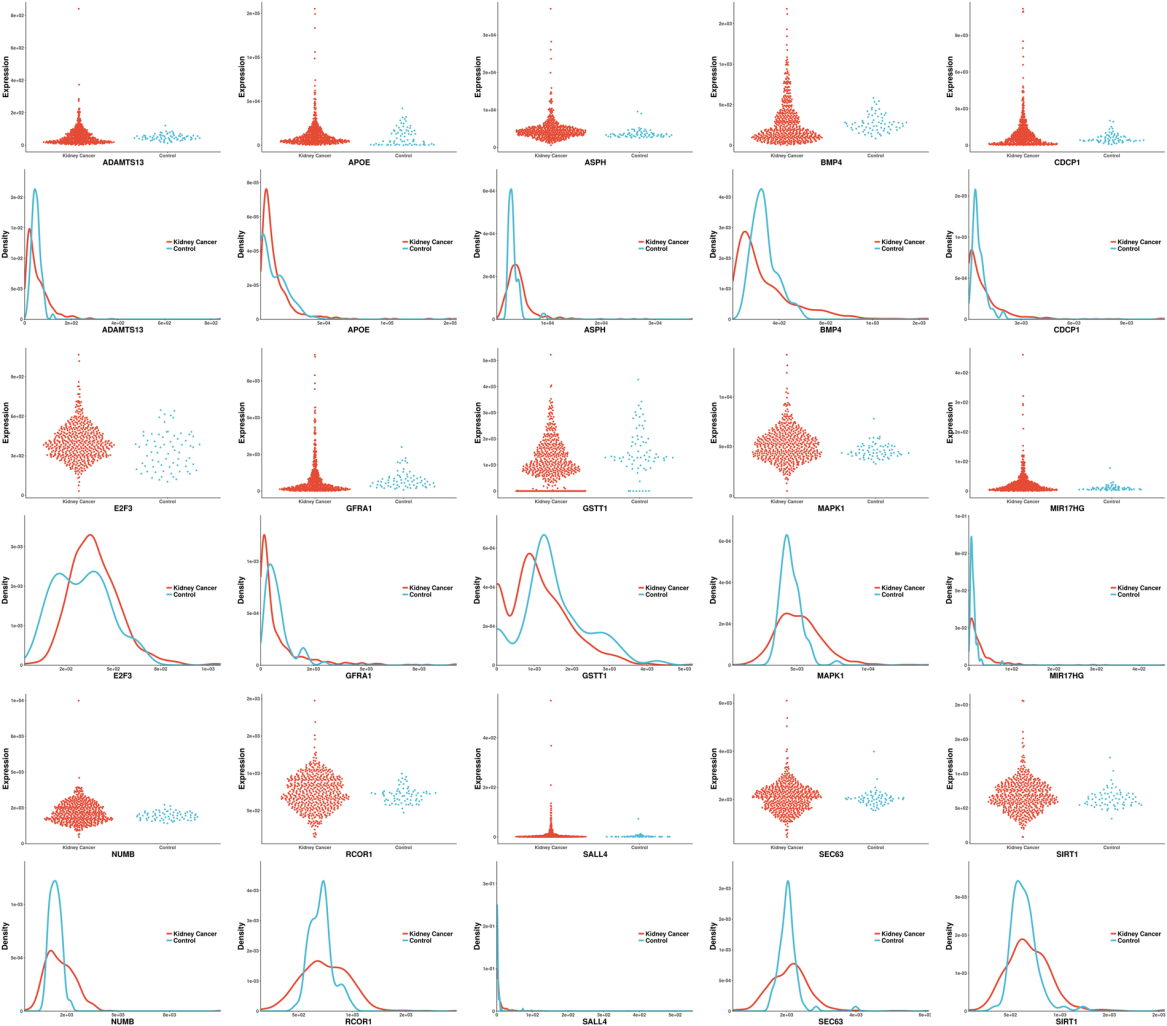
The scatter lot and probability density distribution of 15 gene expressions (reported significant genes detected only by BNNPT) between kidney-cancer and normal groups.

**Figure 2:**
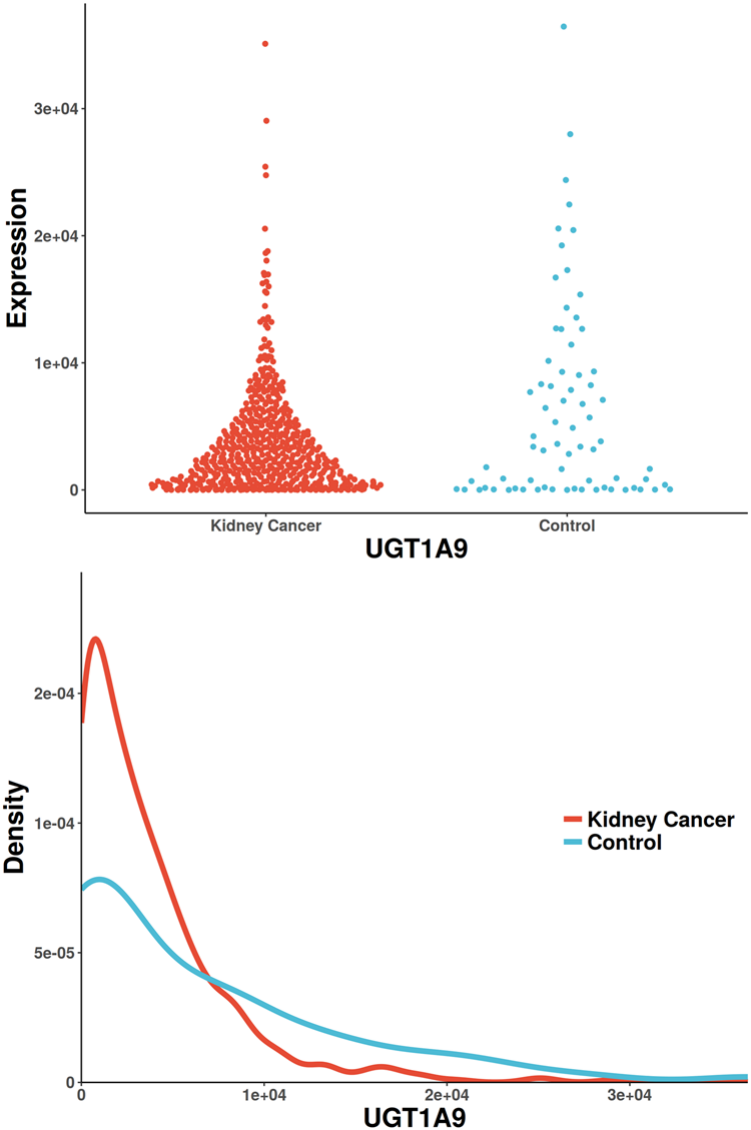
The scatterplot and probability density distribution of UGT1A9 gene expression (reported significant genes detected only by CANOVA) between kidney-cancer and normal groups.

**Figure 3:**
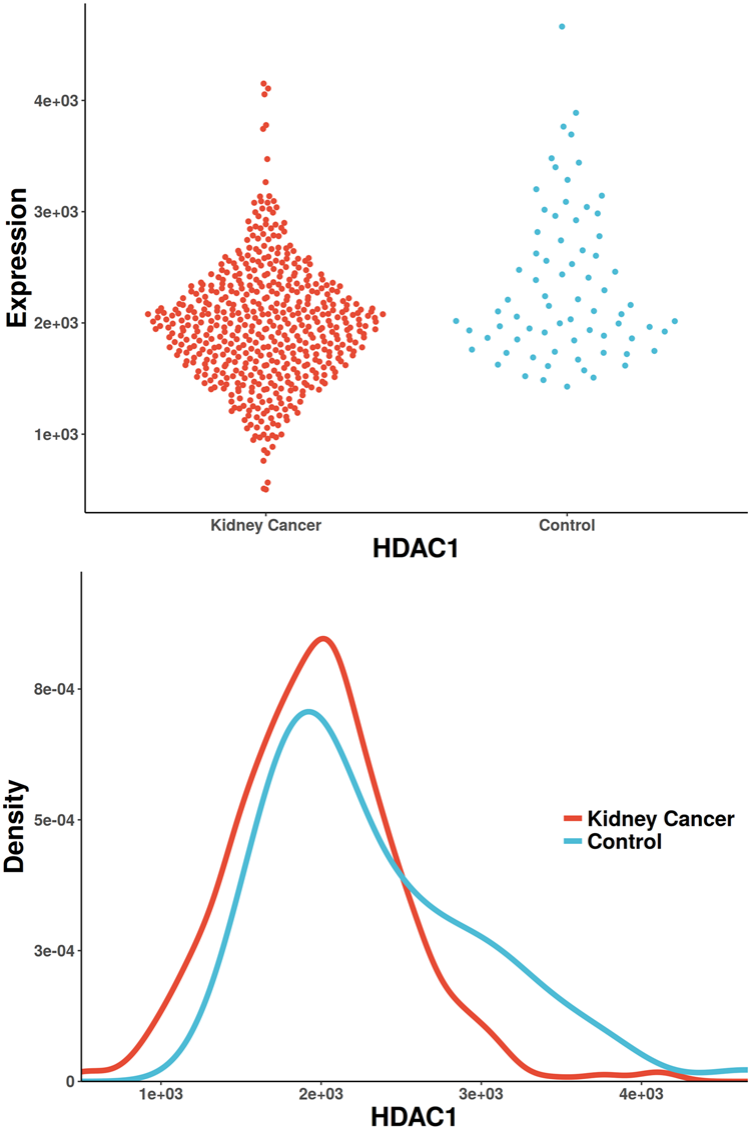
The scatterplot and probability density distribution of HDAC1 gene expression (reported significant genes detected only by Pearson) between kidney-cancer and normal groups.

**Figure 4:**
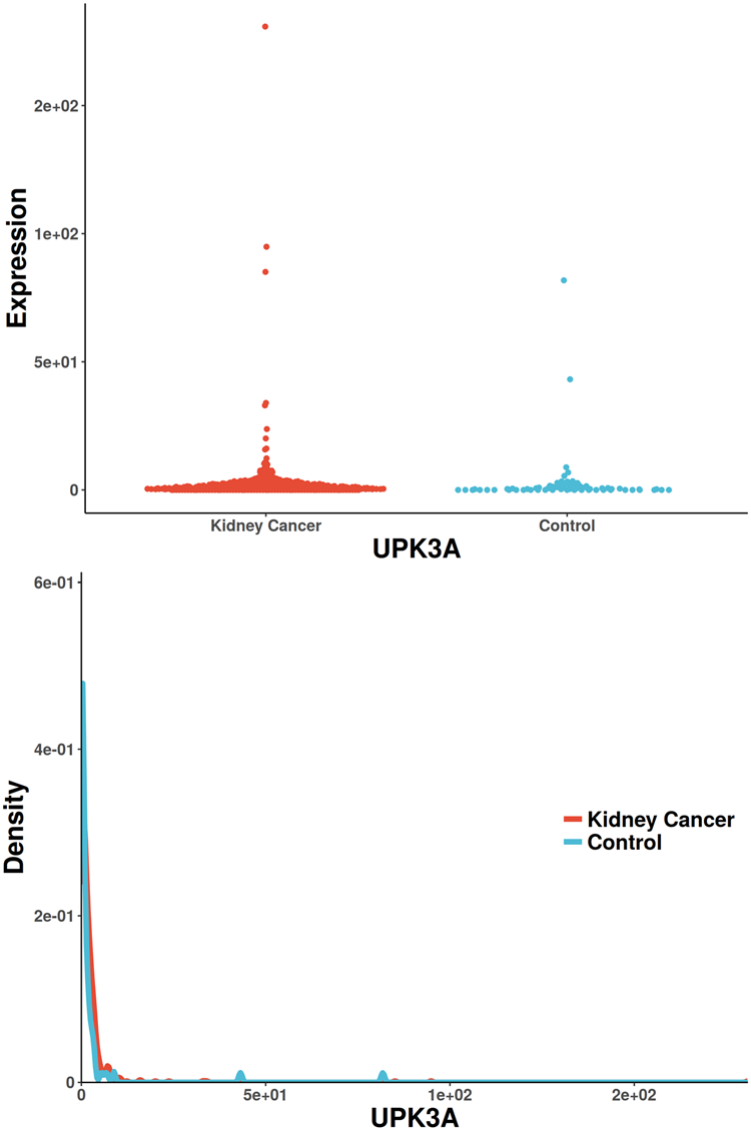
The scatterplot and probability density distribution of UPK3A gene expression (reported significant genes detected only by Spearman) between kidney-cancer and normal groups.

**Figure 5:**
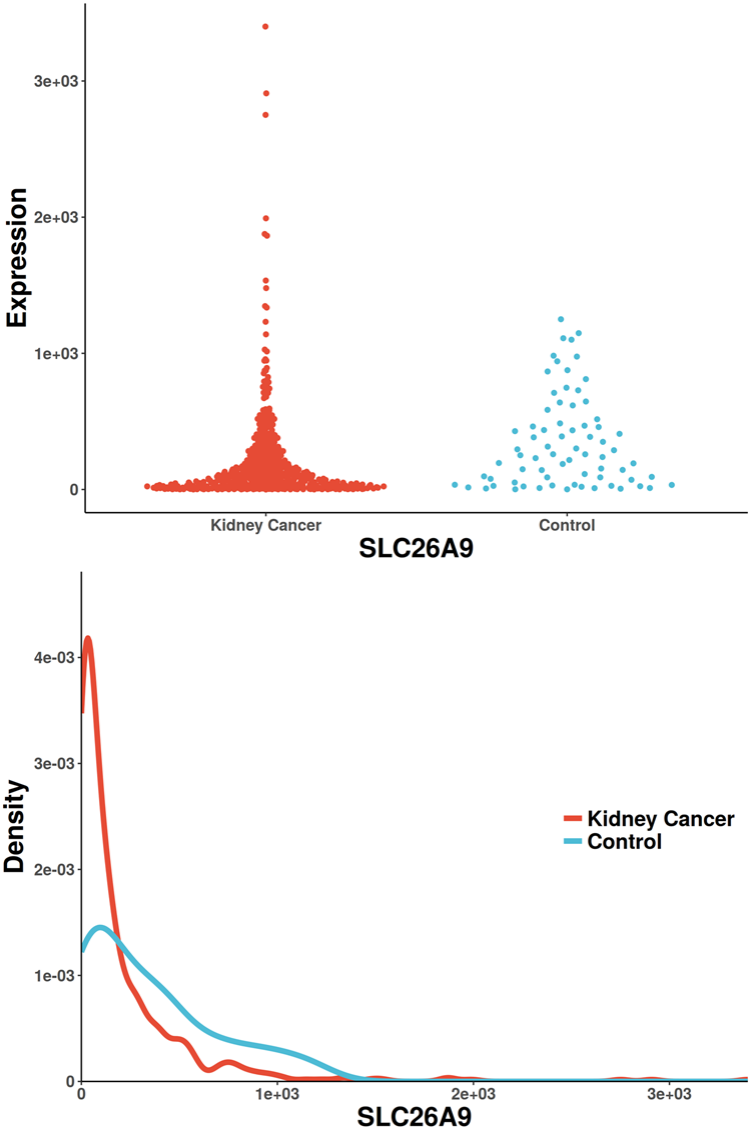
The scatterplot and probability density distribution of SLC26A9 gene expression (reported significant genes detected only by Distance) between kidney-cancer and normal groups.

The uniquely significant genes detected by BNNPT and the corresponding p values of all methods are provided in Supplemental Materials 2, and these genes reported in pubmed (indicating that there is an abstract in pubmed concerning a relationship with kidney cancer and the gene) are shown in Table 4 and Fig. 1 (Scatterplot and probability density distribution). Similarly, the uniquely significant genes found by other methods are shown in Supplemental Materials 3 and the genes reported in pubmed are showed in Figs 2, 3, 4 and 5.

**Table 4:** Reported significant genes detected only by BNNPT and corresponding p-value (the rank of the p-value of each gene from each method) of all methods in kidney cancer dataset (α = 2.435e-06).

Gene	BNNPT	Pearson	Spearman	Kendall	Hoeffding	Distance	CANOVA	MIC*
***APOE***	0.0E + 00 (1)	2.9E-01 (16358)	7.7E-02 (16536)	7.7E-02 (16537)	5.5E-01 (15039)	2.0E-02 (16518)	1.6E-05 (6180)	2.2E-01 (8577)
***ASPH***	0.0E + 00 (1)	3.8E-03 (11660)	1.0E-05 (12081)	1.2E-05 (12081)	3.4E-02 (11141)	6.0E-06 (11295)	3.7E-02 (9785)	2.2E-01 (8606)
***BMP4***	0.0E + 00 (1)	9.3E-01 (19986)	2.1E-03 (14118)	2.2E-03 (14119)	6.1E-02 (11730)	6.4E-05 (12684)	2.4E-02 (8847)	2.1E-01 (9771)
***MIR17HG***	0.0E + 00 (1)	9.1E-04 (10773)	3.5E-05 (12478)	3.9E-05 (12478)	3.9E-02 (11281)	8.0E-06 (11504)	4.5E-02 (10423)	2.1E-01 (9539)
***NUMB***	0.0E + 00 (1)	7.0E-03 (12077)	1.8E-02 (15348)	1.8E-02 (15348)	1.5E-01 (12762)	6.0E-06 (11295)	6.2E-02 (12087)	2.2E-01 (8104)
***RCOR1***	0.0E + 00 (1)	1.9E-01 (15499)	1.8E-01 (17402)	1.8E-01 (17402)	4.0E-01 (13963)	1.0E-04 (12905)	3.9E-02 (9979)	2.2E-01 (8193)
***SEC. 63***	0.0E + 00 (1)	4.8E-01 (17676)	2.0E-01 (17561)	2.0E-01 (17561)	2.2E-01 (13232)	4.6E-05 (12509)	3.9E-02 (9912)	2.0E-01 (9969)
***ADAMTS13***	1.0E-07 (9294)	3.7E-01 (16940)	8.0E-02 (16576)	8.0E-02 (16576)	1.8E-01 (13012)	1.8E-04 (13225)	4.2E-02 (10223)	2.1E-01 (9719)
***CDCP1***	1.0E-07 (9294)	3.4E-02 (13319)	9.3E-01 (20080)	9.3E-01 (20080)	4.8E-01 (14582)	1.1E-03 (14355)	4.8E-02 (10731)	1.9E-01 (11391)
***MAPK1***	2.0E-07 (9613)	3.1E-03 (11494)	1.8E-03 (14056)	1.9E-03 (14056)	1.1E-01 (12409)	6.0E-06 (11295)	7.1E-02 (12898)	2.2E-01 (8447)
***SIRT1***	2.0E-07 (9613)	1.2E-01 (14807)	8.2E-02 (16593)	8.2E-02 (16593)	5.1E-01 (14831)	7.9E-04 (14140)	8.8E-02 (13866)	2.1E-01 (9788)
***E2F3***	2.0E-07 (9613)	7.2E-05 (9558)	2.3E-04 (13181)	2.5E-04 (13181)	3.7E-01 (13847)	1.2E-05 (11758)	3.5E-02 (9659)	1.9E-01 (12135)
***GFRA1***	4.0E-07 (9878)	7.8E-01 (19403)	6.4E-05 (12689)	7.0E-05 (12689)	5.6E-02 (11628)	1.2E-03 (14469)	6.5E-02 (12355)	2.0E-01 (10651)
***GSTT1***	8.0E-07 (10173)	1.3E-04 (9806)	7.9E-05 (12754)	8.6E-05 (12754)	1.6E-01 (12867)	5.2E-05 (12582)	6.0E-02 (11834)	1.8E-01 (12571)
***SALL4***	8.0E-07 (10173)	2.7E-02 (13117)	6.6E-05 (12698)	7.2E-05 (12698)	9.7E-02 (12270)	3.1E-04 (13552)	1.1E-01 (14453)	2.0E-01 (10794)

*As the p-value of MIC is calculated by table lookup, so we just list the MIC value (if MIC > 0.22378, then the p-value of MIC < 2.435e-06). The genes reported in pubmed was shown in bold italics. The rank of the p-value of each gene from each method were also shown above and the ties of p-value ranks were replaced by their minimum respectively.

Out of the unique set of genes detected by BNNPT (Supplemental Materials 3), a few were reported to be relevant to renal cancer or disease: CDCP1, GSTT1, E2F3, MAPK1, SALL4, SIRT1, ADAMTS13, Gfrα1, ASPH, MIR17HG, APOE, BMP4, RCOR1, NUMB and SEC63 (Table 4, Fig. 1). CUB-domain-containing-protein-1 (CDCP1) is an integral membrane protein whose expression is up-regulated in various cancer types. And high CDCP1 expression has been correlated with poor prognosis in renal cancer^39,40^. Emerging evidences suggest that the GSTT1 gene is involved in the detoxification of carcinogens, and the polymorphisms in this gene that result in a loss of enzyme activity may increase the risk of renal cell carcinoma (RCC)^41^. The E2F3 transcriptional regulatory pathway plays an important role in clear cell renal cell carcinoma (ccRCC). E2F3 regulates the carcinogenesis and progression of ccRCC by regulating the expression of downstream HIF-2α^42^. The rs743409 variant in MAPK1 is a variation in the microRNA (miRNA) binding site of the gene in the VHL-HIF1 alpha pathway, which was reported to be significantly associated with renal cell carcinoma^43^. SALL4 is a zinc finger structure transcription factor that maintains the pluripotent of embryonic stem cells and plays an important role in kidney development, its expression is associated with Wilms tumors^44^. SIRT1, acts as a direct target gene for miR-22, significantly inhibits the growth and metastasis of renal cell tumor^45^. The ADAMTS13 gene encodes von Willebrand factor-cleaving protease. It has been reported that human renal tubular epithelial cells synthesize biologically active ADAMTS13 which may, after release from tubuli, regulate hemostasis in the local microenvironment^46^. Gfrα1, combined with tyrosine kinase Ret, is involved in the signaling pathway activated by glial cell line-derived neurotrophic factor (Gdnf), which plays an important role in kidney development and urinary tract maturation^47^. ASPH has been reported to be associated with Congenital anomalies of the kidneys and urinary tract (CAKUT), which are the leading cause of chronic kidney disease (CKD) in children^48^. MIR17HG plays an important role in renal development, especially in the regulation of nephron development, its mutation may affect the renal function^49^. The APOE gene has been reported to be indirectly associated with chronic kidney disease. Knockout of APOE causes hypercholesterolemia, which in turn leads to chronic kidney disease^50^. Mutations in BMP4 are associated with renal abnormalities^51^. RCOR1 and NUMB are associated with renal fibrosis^52,53^. SEC63 is associated with polycystic kidney disease^54,55^.

The mean renal cancer distribution and the normal group distribution are approximately equal for most of the genes in Fig. 1, indicating that the linear relationship is nearly zero (for example, ADAMTS13, APOE, BMP4, GFRA1, RCOR1, SEC63, SIRT1, Pearson R’s p value > 0.05 in Table 4). BNNPT may provide sufficient power if the distributions of these genes have the same mean value, but have different curvature of the density distribution function, meaning that the variances of the two distributions are different. BNNPT is still capable of distinguishing between kidney cancer and normal groups under complex distributions, such as the bimodal distribution in E2F3, to identify the target gene.

The only gene uniquely detected by CANOVA has been reported to be associated with renal cell carcinoma, UGT1A9 (identified in Supplemental Materials 3, Fig. 2). It was reported that a significant decreased glucuronidation capacity was paralleled by drastically reduced UGT1A9 mRNA and protein expression. UGT1A9 mediated renal drug metabolism process, which greatly reduced the incidence of renal cancer^56,57^. There is only one unique gene detected by Pearson (also reported in Pubmed), HDAC1 (identified in Supplemental Materials 3, Fig. 3). The increased activity of histone deacetylase (HDAC) is associated with aggressive tumor behavior and tumor growth. It has been reported that Class I HDAC isoforms 1 and 2 are highly expressed in renal cell cancer^58^. The only unique gene detected by Spearman (also reported in Pubmed) is UPK3A (identified in Supplemental Materials 3, Fig. 4). It has been reported to be associated with vesico-ureteral reflux (VUR), which resulted in 8.5% of end-stage renal disease in children^59^. The only unique gene detected by Distance (also reported in Pubmed) is SLC26A9 (identified in Supplemental Materials 3, Fig. 5), which was reported to be associated with renal disease. SLC26A9 plays an important role in maintaining acid-base balance in renal tubules and nephrons as a chloride ion exchanger^60^. MIC didn’t find unique genes that were previously reported. Hoeffding’s independence test and Kendall’s rank correlation coefficients did not detect any unique significant genes.

## Discussion

Longevity is a multifactorial trait with a genetic contribution and mitochondrial DNA (mtDNA) polymorphisms were found to be involved in the phenomenon of longevity. In an autopsy study of 1,536 patients in Japanese elderly, haplogroups A and M7a were significantly associated with coronary atherosclerosis, with odds ratios (95% confidence intervals) of 1.80 (1.09-2.97; p = 0.023) and 1.92 (1.23-3.01; p = 0.004) respectively^37^. In the study of a population-based case-control study in a Chinese Han population residing in Rugao, Jiangsu Province, a significantly decreasing trend of B4a frequency was observed from middle-aged subjects (4.2%), elderly subjects (3.8%) and longevity subjects (1.7%) in females (p = 0.045). What’s more, significant reduction of M9 haplogroups was observed in longevity subjects (0.2%) when compared with both elderly subjects (2.2%) and middle-aged subjects (1.7%). Linear-by-linear association test revealed a significant decreasing trend of N9 frequency from middle-aged subjects (8.6%), elderly subjects (7.2%) and longevity subjects (4.8%) (p = 0.018)^27^.

Among all the benchmarked methods, BNNPT detected a unique set of genes (15 genes) related to renal cancer or renal diseases in Pubmed database. It was reported that CDCP1 is a unique HIF-2α target gene involved in the regulation of cancer metastasis and suggest that CDCP1 is a biomarker and potential therapeutic target for metastatic cancers^39^. GSTT1 null genotype is a risk factor for patients with more primitive urologic malignancies (bladder, prostate and kidney) and it is more frequent in patients with multiple urologic tumors^41^. Clinical trials have shown that E2F3 is overexpressed in advanced clear cell renal cell carcinoma (ccRCC), and there are multiple E2F3 binding sites in the promoter of HIF-2a. Thus, targeting E2F3-HIF-2a interactions may be a promising treatment procedure for ccRCC^42^. The SNP rs743409 in MAPK1 is a variant of miRNA binding site single nucleotide polymorphisms (SNPs). Under the additive model, the variants were reduced with a 10% risk, indicating that there is a correlation between the miRNA binding site SNP and the RCC risk in the VHL-HIF1 alpha pathway^43^. SALL4 is a zinc finger transcription factor that plays an important role in kidney development, and SALL4 mutation causes kidney deformity^44^. SIRT1 was identified as a direct target for miR-22, and miR-22 might act as a tumor suppressor in RCC and blocks RCC growth and metastasis by direct targeting of SIRT1, indicating a potential new therapeutic effect in RCC therapy^45^. The ADAMTS13 mRNA encodes the von Willebrand factor cleavage protease, which has been detected in a variety of tissues including the kidney. Human renal tubular epithelial cells synthesize ADAMTS13 with biological activity that regulates local microenvironment after release from tubules^46^. Gfrα1 regulates renal development and ureteral maturation in the interaction with the tyrosine kinase Ret and the ligand glial cell-line derived neurotrophic factor (Gdnf)^47^. Other unique genes (ASPH, MIR17HG, APOE, BMP4, RCOR1, NUMB and SEC63) detected by BNNPT are associated with renal diseases^48–55^.

Theoretically, any machine learning algorithm^61^ that predicts Y using X may become the kernel function^62^ of our permutation test. Previously, CANOVA can be viewed as a permutations test of a simple moving average machine learning algorithm. We also tested a random forest^63^ as the kernel, however, both are not as powerful as BNNPT. We speculate that the reason for BNNPT’s superiority is that kNN is the most powerful method in one dimensional machine learning case. Further, we make use of machine learning methods to solve correlation analysis problems.

One important advantage of BNNPT over CANOVA is that the bandwidth parameter can be left as default in most cases. The experiment demonstrates that mtry = sqrt(N) is robust. Thus our test can be viewed as “tuning free”. Also setting mtry = sqrt(N) instead of N (the conventional one nearest neighbor rule) is not only faster but also more powerful due to regularization effect (decorrelation among bags). BNNPT is also robust with the other parameter, number of bags (default is 256 for computing efficiency).

In this study, we can only test independence between two continuous variables. We can’t directly make covariates adjustments. However, we can further take covariate adjustments incorporate into account by first regressing response variable on covariates and then test the independence between the residual error and the response variable Y using BNNPT.

Typically when there exist nonlinear correlation between two variables, the appropriate data transformation can efficiently bring the nonlinearity to linear. We have compared the power of different methods by transforming the data first (including quadratic function, sine function and cosine function in Supplemental Materials 4). And Pearson correlation coefficient is the most powerful method using this strategy. In practice, the true relationship is typically not complex. A two-dimensional scatter plot can help us to reveal the relationship between two variables followed by appropriately chosen transformation model such as log, square or square root transformation. Furthermore, automatically finding the optimal data transformation model is a promising research direction which we will work on in the near future. However, BNNPT is still an efficient method to explore the nonlinear relationships between two continuous variables without specific domain knowledge. According to the null hypothesis, if X really have prediction ability for Y, this dependence could be detected by BNNPT. Moreover, we will also develop multivariable test which may be important on complex traits area^64^.

While each method has its own advantages, the results of different methods can often be correlated with each other. Our simulation results indicate that the use of both linear correlation algorithms (Pearson, Spearman or Kendall) and non-linear correlation algorithms (BNNPT, CANOVA, MIC, Hoeffding or Distance) could increase the probabilities of detecting real biological signals.

To sum, we developed a robust algorithm to detect independence between two random variables especially in non-linear situations. To conclude, our BNNPT method appears to be efficient in testing nonlinear correlation in real data applications.

## Electronic supplementary material

Supplemental Materials 1

Supplemental Materials 2

Supplemental Materials 3

Supplemental Materials 4
